# Effect of *CYP1A1* gene polymorphism and psychological distress on seminal analysis parameters

**DOI:** 10.1186/s12978-016-0169-1

**Published:** 2016-05-24

**Authors:** Aditi Singh, Bidhan Chandra Koner, Prakash Chandra Ray, Sudha Prasad, Elvia Jamatia, Mirza Masroor, Vijay Kumar Singh

**Affiliations:** Department of Biochemistry, Maulana Azad Medical College, New Delhi, 110002 India; Department of Gynaecology and Obstetrics, IVF and Reproductive Biology Centre, Maulana Azad Medical College and Lok Nayak Hospital, New Delhi, India

**Keywords:** Male infertility, Psychological distress, *CYP1A1* polymorphism, Sperm characteristics

## Abstract

**Background:**

Psychological factor alters fertility hormones and contributes to male infertility. Anxiety and depression are common manifestations of psychological distress. Cytochrome P-4501A1 (CYP1A1) metabolizes xenobiotics and fertility hormones that influence male fertility. The effect of *CYP1A1* polymorphism on male fertility has remained controversial.

The present study was designed to assess the effect of psychological distress and *CYP1A1* polymorphisms and their interactions on parameters of seminal analysis.

**Methods:**

Eighty male partners of infertile couples were evaluated for level of distress using Hospital anxiety and depression score (HADS) questionnaire. As per WHO guidelines (2010), sperm count, motility and morphology were assessed and subjects were classified as (a) subjects having normal sperm characteristics and (b) subjects having abnormal sperm characteristics. *CYP1A1* polymorphisms were detected by ASO-PCR.

**Results:**

The significant odd’s ratio indicates that psychological distress (OR:10.54; CI:3.72–29.84; *P* < 0.001), CYP1A1*4(OR:10.31; CI:3.01–35.24; *P* < 0.001) and CYP1A1*2C (OR:7.01; CI:1.78–27.56; *P* = 0.002) polymorphisms are risk factors for the development of abnormal sperm characteristics in male subjects. Data analysis with two way ANOVA shows that psychological distress, CYP1A1*4 and CYP1A1*2C polymorphisms significantly affect but do not interact among them to influence sperm parameters.

**Conclusions:**

It is concluded that *CYP1A1* gene polymorphisms and psychological distress act independently but do not interact with each other in pathogenesis of male infertility.

## Background

Infertility is defined as failure to conceive after 1 year of regular unprotected intercourse with the same partner. Incidence of infertility in India is 10–15 %, as per WHO study and male infertility contributes to 50 % of the total infertile cases [1]. More than 90 % of male infertility cases are due to low sperm counts, poor sperm quality or both.

Psychological factor is closely related to problem of male infertility. Psychological factor is reported to suppress hypothalamic–pituitary–gonadal (HPG) axis through inhibition of gonadotropin-releasing hormone (GnRH) secretion and thereby suppresses luteinizing hormone (LH) release from the pituitary [2]. Contrary to this finding, recently we reported that the effect of stress is more direct on serum testosterone level and the effect on hypothalamus and pituitary is secondary to low serum testosterone level [3]. Zigmond and Snaith developed Hospital anxiety and depression score (HADS) to evaluate psychological distress in the form of anxiety and depression in patients who are not suffering from major psychiatric illness [4] and was later validated by Bjelland et al. through a systematic review of large number of studies [5]. HADS is widely used for assessing psychological distress among the infertility cases [3, 6].

Cytochrome P-4501A1 (CYP1A1) is an inducible microsomal enzyme encoded by the *CYP1A1* gene. Single nucleotide polymorphism (SNP) of *CYP1A1* gene is known to interfere with the activity of CYP1A1 enzyme. It is a phase 1 xenobiotic catabolizing enzyme that acts on polyaromatic hydrocarbons (PAHs) [7]. Many of these PAHs are endocrine disrupters and their CYP1A1 activated metabolite can form DNA adduct. DNA adducts in sperm cells could be considered as a sign of severe DNA damage, which played an important role in meiotic division during spermatogenesis and could be associated with male infertility [8]. Besides the xenobiotic metabolizing action, CYP1A1 is also responsible for estrogen and testosterone metabolism [9–11]. CYP1A1 is involved in hydroxylation and biotransformation of these hormones [10]. This altered CYP1A1 activity might be implicated in male fertility process. Vani et al. [12] genotyped CYP1A1*2A using PCR RFLP assay in a hospital based case control study and showed a significant risk of infertility among the male subjects carrying CYP1A1*2ACC genotype. Aydos et al. [13] conducted a study to see the relationship between genetic polymorphism of CYP1A1*2C and male factor infertility. A person having *CYP1A1*val/val or *CYP1A*1lle/lle along with Glutathione S transferase M i.e., GSTM null genotype was shown to have 6.9 times more risk of infertility than a person having *CYP1A*1lle/lle along with GSTM1 wild type genotype. They concluded an important role of genetic polymorphisms of xenobiotic metabolizing enzymes in male infertility [13]. In a study from China (2007), no significant association was detected between CYP1A1*2A polymorphism and male infertility [14]. So there are contradictory reports on the role of *CYP1A1* polymorphism on male infertility.

Psychological factor and *CYP1A1* single nucleotide polymorphisms (SNPs) might separately act as a risk factor for male infertility. SNPs of *CYP1A1* and psychological distress can alter testosterone and estrogen levels and enhance oxygen free radical formation and thus share common mechanisms to induce infertility in men. So it is possible that they interact with each other in precipitating male infertility. The present study is to explore the role of *CYP1A1* polymorphism and psychological factor and their interaction, if any, in pathogenesis of development of abnormal sperm characteristics.

## Method

### Study subjects

The study was conducted on 80 adult male subjects (age: 27 ± 3.08 years, range: 20–35 years; Duration of infertility:2–8 years). Male partner of infertile couples attending infertility clinic were selected randomly (1–2 cases per day). The subjects who were advised by the treating physician for a semen analysis for the primary infertility problem, were found normal on physical examination and gave written consent for being incorporated in this research work were included in this study. The persons with defect in seminal analysis as defined by WHO [15] were considered as subjects with abnormal sperm characteristics (age: 27 ± 3.02 years, range: 22–34 years; Duration of infertility:2–7 years) and the rests (age: 28 ± 3.19 years, range: 20–35 years; Duration of infertility:2–8 years) were having normal seminal character in this study. The subjects with known infections, any major heart, lungs, liver and kidney diseases, presence of WBCs in the semen, erectile dysfunction, congenital genital abnormality, hydrocele, crypotorchidism and varicocele were excluded from the study. It was a hospital based cross sectional study and was approved by institutional ethical committee (Ref. no. F.11/IEC/MAMC/10/No.42). The work was carried out in the Department of Biochemistry in collaboration with IVF and Reproductive Biology Centre, Department of Gynaecology and Obstetrics, LN Hospital, New Delhi.

### Assesment of psychological distress

Subjects were evaluated for anxiety symptoms and depression for screening psychological distress using the HADS [16]. It is a 14 item screening scale that was originally developed to indicate the possible presence of anxiety and depression states during the past 2 weeks in the setting of a medical non-psychiatric outpatient clinic. The HADS is comprised of two 7 item subscales, one for anxiety (HADS-A) and other for depression (HADS-D). Each item scores on a 4-point likert scale ranging from 0 (not at all) to 3 (nearly every day), giving maximum subscale scores of 21 for depression and anxiety, respectively. Higher scores indicate greater likelihood of depression or anxiety. Recommended cut offs are: Normal: 0–7, mild cases: 8–10, moderate cases: 11–15 and severe cases:16 or above. It is written in English, translated by us in Hindi and Hindi version was validated. But as most of the patients were illiterate and had difficulty in reading and comprehending even the translated version of self-rating HADS questionnaire, so it was administered in the form of a structured interview as done in our previous studies [3]. As the HADS is easy to score and administer, it is used as a screening instrument for psychological distress.

### Estimation of seminal parameters

Seminal analysis was performed as per WHO guidelines, 2010 [15]. It was carried out by a trained technician dedicated for this purpose only at IVF centre, Lok Nayak Hospital. In brief, semen obtained by masturbation after 2–6 days of sexual abstinence and ejaculated into a wide-mouthed plastic container was used for analysis. Verbal and written instructions were given to the subjects before semen collection. The volume of semen was measured by weighing the sample and multiplying its weight by the density (1 gm ml^-1^) of semen. For calculating sperm concentration, well-mixed liquefied semen was suitably diluted with fixative and mounted in a 100- μm-deep haemocytometer chamber. At least 200 intact spermatozoa per replicate were counted. The total number of spermatozoa per ejaculate was calculated by multiplying sperm concentration with semen volume. Sperm motility was assessed after liquefaction of semen at room temperature within 1 h of ejaculation from a 20-μm-deep wet preparation examined under phage-contrast microscope at 9400 magnification. At least 200 spermatozoa were evaluated in a total of at least five fields in at least two replicates to achieve acceptably low sampling error for motility assay. The motility of each spermatozoon was graded as follows: (i) progressive motility: where the spermatozoa moves actively either linearly or in a large circle, (ii) non progressive motility: where the spermatozoa are motile with an absence of progression and (iii) immotility: where the spermatozoa are not moving. Normal and abnormal morphology were assessed as defined in the WHO manual (2010) that includes Kruger’s criteria. Assessment of leucocytes in semen was carried out by staining cellular peroxidase using ortho-toluidine as described in WHO manual (2010). Semen having 1X10^6^ or more peroxidase-positive cells was excluded from the study. A person was considered to have abnormal sperm characteristics when his sperm characteristics deviated from cut off limit as defined by WHO [15]. Seminal analysis report is considered normal when semen volume was >2 ml/ejaculation and/or liquified completely within 60 min at room temp and/or sperm count is 20million/ml of semen or 40million/ejaculate and/or motility is 50 % or more with forward progression and 25 % or more with rapid progression within 60 min of collection and/or morphology is 15 % or more within normal forms and/or leukocyte count is <1million/ml of semen.

### Gene polymorphism

Allele-specific PCR method was used as a mutation detection method. Total genomic DNA was isolated from whole blood using Genomic DNA Minikit (Geneaid, Taiwan). The instructions of manufacturer were followed to extract DNA. The presence of DNA in the extract was confirmed by Agarose gel electrophoresis. PCR was carried out in a total volume of 25 μl (10 μl of mastermix, 0.3 μl of forward primer, 0.3 μl of reverse primer, 12.5 μl of nuclease free water and 2 μl of total genomic DNA) using thermocycler (Bioer, China).

Primers used for CYP1A1*4 (rs1799814; 2453C > A) polymorphism were:

Forward: For CC allele- 5′AAGCGGAAGTGTATCGGTGAGACC 3′

For AA allele- 5′AAGCGGAAGTGTATCGGTGAGAAC 3′

Reverse: 5′CAGGTAGACAGAGTCTAGGCCTCAG 3′

Primers used for CYP1A1*2C (rs 1048943; 2455 A > G) polymorphism were:

Forward: For AA allele- 5′GGAAGTGTATCGGTGAGACCA 3′

For GG allele- 5′GGAAGTGTATCGGTGAGACCG 3′

Reverse: 5′TCATGTCCACCTTCACGCCCA 3′

PCR conditions for CYP1A1*4 (rs1799814; 2453C > A) polymorphism were: Initial denaturation at 94 °C for 10 min, 40 cycles each of denaturation at 94 °C for 40 s, annealing at 62 °C for 40 s, extension at 72 °C for 35 s and final extension at 4 °C for 10 min followed by storage at 4 °C for 10 min.

PCR conditions for CYP1A1*2C (rs 1048943; 2455 A > G) polymorphism were: Initial denaturation: 94 °C for 10 min, 40 cycles each of: denaturation at 94 °C for 40 s, annealing at 59.9 °C for 40 s, extension at 72 °C for 35 s and final extension at 4 °C for 10 min followed by storage at 4 °C for 10 min.

Amplified products were electrophoresed on 2 % agarose gel to detect the products of 218 bp for CYP1A1*4 (rs1799814; 2453C > A) polymorphism and 108 bp for CYP1A1*2C (rs 1048943; 2455 A > G) polymorphism.

STATISTICAL ANALYSIS: Odd’s ratio was calculated to estimate the risk of distress and gene polymorphism for development of abnormal sperm characteristics. Significance of odd’s ratio was calculated by using Chi-square/Fisher exact test. For comparison of data, Mann Whitney *U* test or Kruskal Wallis test was used. Two way ANOVA (univariate analysis of variance) was performed to evaluate the effect of and interactions among independent factors (distress and gene polymorphisms) on sperm characteristics. A *P* value <0.05 was considered as statistically significant in all statistical analysis.

## Result and discussion

The present study was designed to evaluate the effect of psychological distress, *CYP1A1* polymorphisms and their interactions in the development of abnormal sperm characteristics. We selected rs 1799814 and rs1048943 of *CYP1A1* because of its high prevalence in the population and role of CYP1A1 in metabolism of fertility hormones and xenobiotic substances/endocrine disrupters, those are known to influence male fertility.

Table 1 shows that the odd’s of having abnormal sperm characteristics associated with psychological distress is 10.54 (CI: 3.72–29.84; *P* <0.001). As abnormal sperm characteristics contributes to 90 % of male infertility, this indicates that psychological distress is a risk factor for male infertility. Previous studies carried out on animals and humans also have demonstrated that stress can cause male infertility [17, 18]. Stress-induced alteration of fertility hormones involving Hypothalamo-Pituitary-Gonadal axis is claimed be the cause stress-induced male infertility [3]. The table also shows that the risk of having abnormal sperm characteristics associated with heterozygocity (CA genotype) of CYP1A1*4 and AG genotype of CYP1A1*2C is 10.31 (CI: 3.01–35.22, *P* <0.001) and 7.01 (CI: 1.78–27.56, *P* = 0.002) times higher respectively than that of their wild homozygote counterparts. It indicates that CYP1A1*4 and CYP1A1*2C polymorphisms are associated with higher genetic risk for development of male infertility. Previous studies have evaluated effect of *CYP1A1* polymorphism on male infertility. In a study from China, no significant association was detected between CYP1A1*2A polymorphism and male infertility [14], but Vani et al., [12] showed a significantly increased risk of male infertility with CYP1A1*2A CC genotype, a non significantly increased risk with TC genotype and a significantly increased risk with both the variant genotypes combined (CYP1A1*2A TC + CC) assuming a co dominant allele effect. Present study evaluated different polymorphisms of *CYP1A1* and it is seen that they are associated with genetic risk of having abnormal sperm characteristics. The difference in the result might be because of selecting different loci of polymorphism of *CYP1A1* gene, study design or final outcome parameters. The outcome parameter of previous studies was infertility but that in the present study was sperm charecteristics. In previous studies, infertile males were cases and fertile subjects were the controls, whereas the present study involved a cross section of male partners of infertile couples irrespective of their fertility potentials. Later on they were divided based on their seminal analysis report. So the study design has overlap of cross-sectional and case-control features. The rise in odd’s associated with mutant homozygocity of CYP1A1*4 and CYP1A1*2C were not significant. This might be because of less number of homozygotes in the sample.

**Table 1:** Risk of development of abnormal sperm characteristics associated with psychological distress (HADS >7), CYP1A1*4 and CYP1A1*2C polymorphism

Factors		Subjects with abnormal sperm characteristics	Subjects with normal sperm characteristics	ODD’s ratio (0.95 confidence interval)
Psychological distress	Distress –ve	8	29	1
	Distress + ve	32	11	10.54 (3.72–29.84)*
CYP1A1*4	CC	4	20	1
	CA	33	16	10.31 (3.01–35.22)*
	AA	3	4	3.75 (0.59–23.66)
CYP1A1*2C	AA	3	13	1
	AG	34	21	7.01 (1.78–27.56)*
	GG	3	6	2.16 (0.33–14.05)

*P < 0.05 by Chi-square test

Table 2 shows that psychological distress significantly affect sperm count [Median(range): 63(2–105) million/ml in subjects without distress Vs 10(0–140)million/ml in subjects with distress], motility [Median (range) in percentage of actively motile sperm: 60(10–74) in subjects without distress Vs 20(0–80) in subjects with distress] and morphology [Median (range) in percentage of morphologically normal sperm: 60(10–98) in subjects without distress Vs 25(2–90) in subjects with distress]. This is consistent with our previous study where we showed correlation of altered sperm parameters with the altered fertility hormones associated with stress [3]. It indicates that psychological distress alters sperm parameters by influencing fertility hormones. Table 3 shows that CYP1A1*4 (rs1799814; 2453C > A) and *CYP1A*1*2C (rs1048943; 2455 A > G) polymorphisms significantly decrease the total sperm count and the percentage of progressively motile sperms in male subjects but their effect on sperm morphology were not statistically significant. Molecular mechanism of this effect is yet to be explored. *CYP1A1* is a gene responsible for xenobiotic and fertility hormone metabolism. Both xenobiotic substances and fertility hormones are known to have influence on male fertility. The risk of infertility attributed to these polymorphisms might be because of altered Cyt P-450 activity leading to altered xenobiotic and steroid hormone metabolism associated with it. Evaluation of the effect of these SNPs on mRNA of *CYP1A1,* Cyt P-450 enzyme activity and fertility hormones can throw more light on this aspect. However, the present study did not assess these parameters.

**Table 2:** Comparison of seminal analysis parameters between subjects without and with psychological distress (HADS >7)

Median (Range) of seminal analysis parameters	Distress -ve	Distress + ve	*P* value
Sperm Count (x 10^6^/ml)	63 (2–105)	10 (0–140)	0.001
Sperm Motility (%)			
a) Progressive motility	60 (10–74)	20 (0–80)	0.001
b) Non-progressive motility	20 (5–50)	20 (0–70)	0.642
c) Immotile	20 (0–76)	30 (0–95)	0.002)
Sperm Morphology (%):			
a) Normal	60 (10–98)	25 (2–90)	0.001
b) Abnormal	40 (2–95)	70 (0–98)	0.003

*P value calculated by Mann Whitney *U* test in comparison to distress negative (–ve) subjects

**Table 3:** Comparison of seminal analysis parameters between subjects with different genotypes of *CYP1A1* gene polymorphisms

	For CYP1A1*4 C > A				For CYP1A1*2C A > G			
	Genotype	N	Median (range)	*P*-value*	Genotype	N	Median (range)	*P*-value*
Sperm Count (x 10^6^/ml)	CC	24	63 (2.5–125)	0.003	AA	16	70 (2.5–125)	<0.001
	AC	49	10 (0–140)		AG	55	20 (0–105)	
	AA	07	45 (1.5–90)		GG	09	62 (5–140)	
Sperm Motility (%)								
Progressive Motility	CC	24	59 (15–95)	0.003	AA	16	63.5 (10–90)	0.016
	AC	49	25 (0–95)		AG	55	30 (0–95)	
	AA	07	40 (10–80)		GG	09	60 (5–78)	
b) Non- progressive	CC	24	20 (5–40)	0.410	AA	16	17.5 (5–70)	0.933
	AC	49	18 (0–70)		AG	55	20 (0–70)	
	AA	07	20 (5–70)		GG	09	20 (0–40)	
c) Immotile	CC	24	20 (0–70)	0.305	AA	16	20 (5–30)	0.290
	AC	49	20 (0–95)		AG	55	25 (0–95)	
	AA	07	25 (5–60)		GG	09	16 (0–70)	
Sperm Morphology								
Normal	CC	24	59 (5–92)	0.074	AA	16	61.5 (5–98)	0.056
	AC	49	32 (0–95)		AG	55	40 (0–90)	
	AA	07	58 (0–98)		GG	09	66 (25–95)	
b) Abnormal	CC	24	49 (8–95)	0.108	AA	16	38.5 (2–95)	0.196
	AC	49	60 (0–98)		AG	55	55 (0–98)	
	AA	07	42 (2–92)		GG	09	34 (5–75)	

*P value calculated by Kruskal wallis test

Tables 4, 5 and 6 depict the outcome of two way ANOVA performed after taking psychological distress, CYP1A1*4 and *CYP1A*1*2C polymorphism as independent variables and sperm count, motility and morphology as dependent variables. Table 4 shows that psychological distress, CYP1A1*4 and CYP1A1*2C polymorphism have got significant effect on sperm count, but psychological distress and the *CYP* SNPs do not interact with each other in decreasing the sperm count. Similarly Table 5 shows that psychological distress, CYP1A1*4 and CYP1A1*2C polymorphism per se influenced the sperm motility but there was no interactions among these factors to decrease the sperm motility. Table 6 depicts that psychological distress and CYP1A1*2C polymorphism per se significantly influenced the sperm morphology but CYP1A1*4 polymorphism did not have any influence on sperm morphology and their were no interactions between psychological distress and the two SNPs in influencing sperm morphology. Although CYP1A1 gene polymorphisms and distress are found to have similar effect on sperm count and motility, their coexistence did not have any synergistic effect on the sperm characteristics.

**Table 4:** Two way ANOVA taking sperm count as dependent variable and psychological distress and *CYP1A1* gene polymorphisms as independent variables (*n* = 80)

	CYP1A1*4 C > A^a^				
	Type III sum of squares	Degree of Freedom	Mean Square	*F* value	*P* value
Corrected Model	36223.220^a^	5	7244.644	07.383	<0.001
Intercept	78599.320	1	78599.320	80.098	0.001
CYP1A1*4 C > A Polymorphism	14681.909	2	340.954	07.481	<0.001
Distress	13708.246	1	13708.246	13.970	<0.001
Interaction of CYP1A1*4 C > A Polymorphism with Distress	3145.427	2	1572.714	01.603	0.208
	CYP1A1*2C A > G^b^				
Corrected Model	42386.59^b^	5	8477.31	09.44	0.000
Intercept	130860.45	1	130860.40	145.72	0.000
CYP1A1*2C A > G Polymorphism	7497.00	1	7497.00	08.34	0.005
Distress	21793.27	2	10896.63	12.13	0.000
Interaction of CYP1A1*2C A > G Polymorphism with Distress	1912.36	2	956.18	01.06	0.350

^a^R Squared = 0.333 (Adjusted R Squared = 0.288). ^b^R Squared = 0.460 (Adjusted R Squared = 0.423)

**Table 5:** Two way ANOVA taking sperm motility (% of progressively motile sperm) as dependent variable and psychological distress and *CYP1A1* gene polymorphisms as independent variable (*n* = 80)

	CYP1A1*4 C > A^a^				
	Type III sum of squares	Degree of Freedom	Mean Square	*F* value	*P* value
Corrected Model	27926.20^a^	5	5585.24	12.58	<0.001
Intercept	84058.13	1	84058.13	189.38	<0.001
CYP1A1*4C > A Polymorphism	7531.78	2	3765.89	08.48	<0.001
Distress	10528.13	1	10528.13	23.72	<0.001
Interaction of CYP1A1*4C > A Polymorphism with Distress	1141.34	2	570.67	01.28	0.283
	CYP1A1*2C A > G^b^				
Corrected Model	25028.29^b^	5	5005.66	10.36	<0.001
Intercept	108286.74	1	108286.74	224.19	<0.001
CYP1A1*2C A > G Polymorphism	5692.88	2	2846.44	05.89	0.004
Distress	12091.25	1	12091.21	25.03	<0.001
Interaction of CYP1A1*2C A > G Polymorphism with Distress	268.55	2	134.27	0.27	0.758

^a^R Squared = 0.460 (Adjusted R Squared = 0.423). ^b^R Squared = 0.412 (Adjusted R Squared = 0.372)

**Table 6:** Two way ANOVA of sperm morphology (% of morphologically normal sperm) as dependent variable, psychological distress and *CYP1A1* gene polymorphisms as independent variables (*n* = 80)

	CYP1A1*4 C > A^a^				
	Type III sum of squares	Degree of Freedom	Mean Square	*F* value	*P* value
Corrected Model	17241.26^a^	5	3448.25	05.30	0.000
Intercept	102868.16	1	102868.16	158.11	0.000
CYP1A1*4C > A Polymorphism	3250.97	2	1625.48	02.49	0.089
Distress	10517.43	1	10517.43	16.16	0.000
Interaction of CYP1A1*4C > A Polymorphism with Distress	1180.37	2	590.18	0.90	0.408
	CYP1A1*2C A > G^b^				
Corrected Model	17897.47^b^	5	3579.45	05.57	0.000
Intercept	129471.69	1	129471.69	201.75	0.000
CYP1A1*2C A > G Polymorphism	4873.00	2	2436.50	03.79	0.027
Distress	8668.33	1	8668.33	13.50	0.000
Interaction of CYP1A1*2C A > G Polymorphism with Distress	408.88	2	204.44	0.31	0.728

^a^R Squared = 0.264 (Adjusted R Squared = 0.214). ^b^R Squared = 0.274 (Adjusted R Squared = 0.225)

## Conclusion

So, it is concluded that *CYP1A1* gene polymorphisms and psychological distress act independently in developing abnormal sperm characteristics and thereby might contribute to pathogenesis of male infertility. But these factors do not interact among them in influencing parameters of seminal analysis despite having their similar effects on steroid hormones that control male fertility.
